# Sperm competition: linking form to function

**DOI:** 10.1186/1471-2148-8-319

**Published:** 2008-11-25

**Authors:** Stuart Humphries, Jonathan P Evans, Leigh W Simmons

**Affiliations:** 1 Department of Animal & Plant Sciences, University of Sheffield, Alfred Denny Building, Western Bank, Sheffield S10 2TN, UK; 2 Centre for Evolutionary Biology, School of Animal Biology (M092), The University of Western Australia, Crawley, WA 6009, Australia

## Abstract

**Background:**

Using information from physics, biomechanics and evolutionary biology, we explore the implications of physical constraints on sperm performance, and review empirical evidence for links between sperm length and sperm competition (where two or more males compete to fertilise a female's eggs). A common theme in the literature on sperm competition is that selection for increased sperm performance in polyandrous species will favour the evolution of longer, and therefore faster swimming, sperm. This argument is based on the common assumption that sperm swimming velocity is directly related to sperm length, due to the increased thrust produced by longer flagella.

**Results:**

We critically evaluate the evidence for links between sperm morphology and swimming speed, and draw on cross-disciplinary studies to show that the assumption that velocity is directly related to sperm length will rarely be satisfied in the microscopic world in which sperm operate.

**Conclusion:**

We show that increased sperm length is unlikely to be driven by selection for increased swimming speed, and that the relative lengths of a sperm's constituent parts, rather than their absolute lengths, are likely to be the target of selection. All else being equal, we suggest that a simple measure of the ratio of head to tail length should be used to assess the possible link between morphology and speed. However, this is most likely to be the case for external fertilizers in which females have relatively limited opportunity to influence a sperm's motility.

## Background

Although several theories regarding the evolution of sperm size exist [for reviews see [1,2]], there is a general assumption in the literature on sperm competition that selection will favour males with longer sperm, due to their enhanced swimming velocity and therefore competitiveness. This assumption rests broadly on three observations. First, a number of comparative studies have reported that sperm are on average longer in polyandrous species compared to monandrous species [e.g. [3-5]]. These evolutionary associations are generally taken as evidence that selection for enhanced sperm competitive ability favours increased sperm length in polyandrous species, where females mate with more than one male during a single reproductive episode and sperm from different males must compete to fertilize available ova [6]. Second, a handful of studies have reported that relative sperm size can be associated with competitive fertilization success [e.g. [7]]. And third, four studies have reported that sperm swimming velocity, and therefore possibly sperm competitiveness, covaries with some measure of sperm length, hinting at a functional relationship between these phenotypic traits [4,8-10].

While the first two of these observations are correlational, the last suggests a mechanistic relationship linking sperm structure to function. However, empirical evidence of such a link is conflicting (Table 1). Explaining why longer sperm might be more competitive than shorter ones, and how selection acts on this difference, is fundamental to our understanding of sperm evolution. Thus, our aim here is to re-evaluate the proposed link between sperm length and swimming speed using theory from physics and biomechanics. We explore the hydrodynamic environment in which sperm operate, which we argue invalidates common assumptions that link sperm shape and length to swimming velocity. We then argue that because of the underlying hydrodynamic interactions governing sperm motion, there is no reason to expect a simple association between sperm length and sperm velocity. Instead, we propose alternative measures that take account of these interactions in an attempt to guide future studies that link sperm competition with sperm evolution. We argue that the focus on sperm speed and length has come at the expense of neglecting alternative mechanisms that can help explain a wider variety of the data available.

**Table 1:** Published relationships between sperm phenotypic traits and swimming speed.

Taxon	Morphological variable	Correlation with speed	Study
Mammals	Total length	+	[4,8]
Red deer (*Cervus elaphus hispanicus*)	Head length	+	[9]
	Midpiece length	-	
	Flagellum length	0	
	Total length	0	
Zebra finch (*Taeniopygia guttata*)	Midpiece length	0	[43]
	Flagellum length	0	
	Tail length	0	
Cichlid fish (*Telmatochromis vittatus*)	Total length	0	[45]
Atlantic salmon (*Salmo salar*)	Head length	0	[46]
	Flagellum length	0	
	Total length	0	
Bluegill sunfish (*Lepomis macrochirus*)	Total length	0	[44,66]
	Flagellum length	0	[42]
Guppy (*Poecilia reticulata*)	Head length	+	[10]
	Flagellum length	0	
	Relative flagellum length	0	
Grass goby (*Zosterisessor ophiocephalus*)	Tail length	0	[67]
	Total length	0	
Black goby (*Gobius niger*)	Tail length	0	[67]
	Total length	0	
Land snail (*Arianta arbustorum*)	Total length	0	[47]

Key: +, positive correlation between length and speed; -, negative correlation between length and speed; and 0, correlation analysis performed, but no relationship between length and speed found.

## Methods and results

### Insight from physics

We argue that the complex physical constraints governing sperm locomotion may obscure simple relationships between sperm length and swimming velocity, thus accounting for the inconsistent patterns of covariance between these traits reported in the literature. To illustrate this, we first explore the hydrodynamic environment in which sperm operate, which we argue invalidates common assumptions that link sperm shape and length to swimming velocity.

#### (a) Shape and drag

The diversity of sperm size and shape is considerable [e.g. [11]], but in general the body plan of a flagellate spermatozoon follows the pattern of a head containing the nuclear material, a midpiece containing mitochondria, and a tail based on the eukaryotic flagellum. Superficially, the shape of the tail and the motion of swimming are eel-like, and it is often assumed that locomotion too works in a similar way. However, small size and low swimming speeds mean that, in hydrodynamic terms, sperm operate in a very different regime from the one that we are used to. This regime can be defined by the use of the Reynolds number, which is equal to the relative ratio between inertia and viscosity. The Reynolds number is given by *Re *= *ul*/*μ*, where *u *is speed, *l *is a characteristic length of the object of interest (conventionally length in the direction of travel, e.g. total sperm length), and μ is the kinematic viscosity of the fluid relative to which the object is moving. In the context of biology, size and speed tend to be positively correlated [12], so that in their natural environments small organisms operate in a low Reynolds number regime (*Re *<< 1)[13].

**Figure 2:**
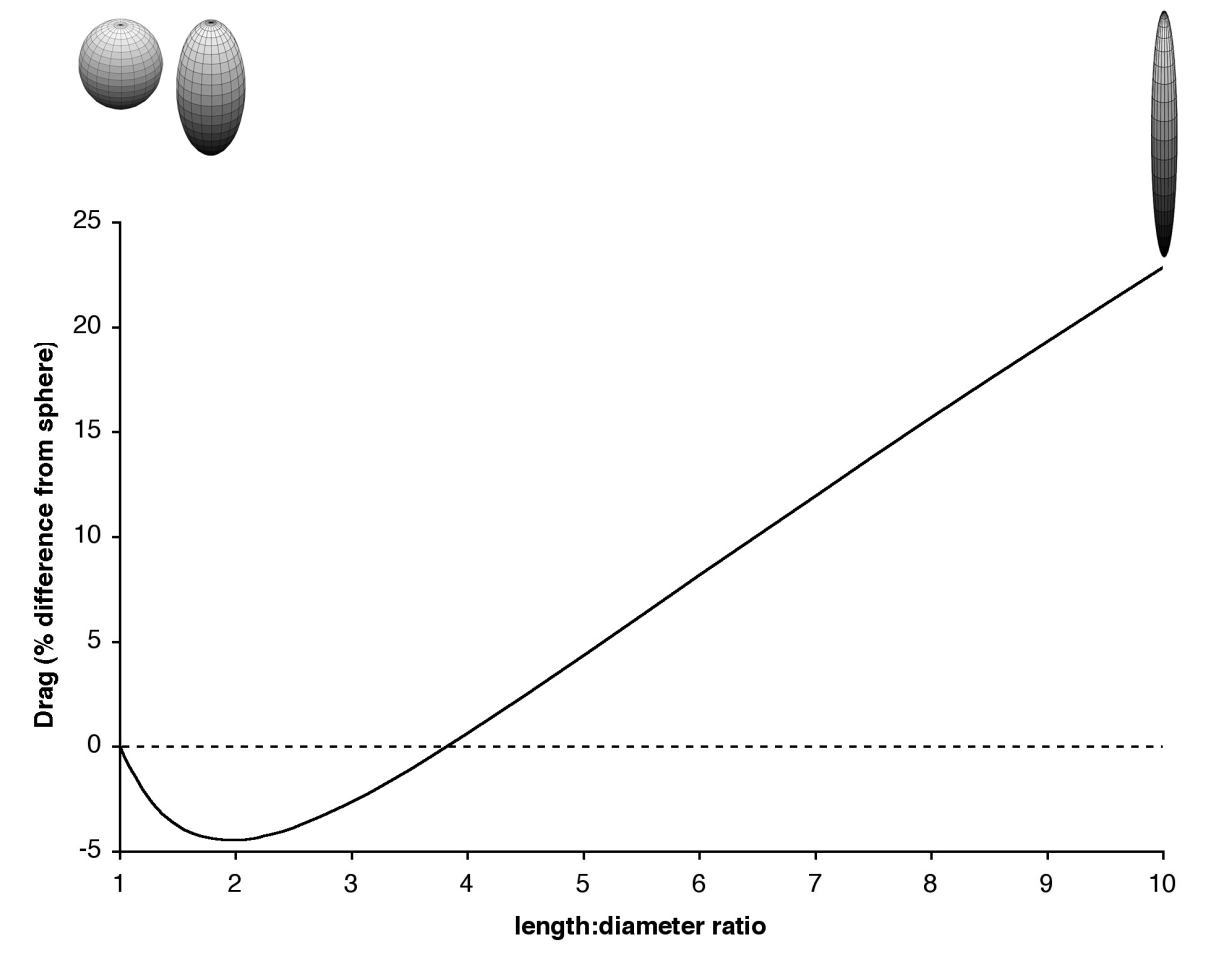
**Percentage difference in drag between a sphere and a prolate spheroid of identical volume at *Re *<< 1**. As the ratio of length to diameter of the spheroid increases (i.e. the shape elongates) there is an initial decrease in drag, but this difference only results in drag for the spheroid dropping to a minimum of 95.66% of that of the sphere.

Streamlining, the modification of shape to reduce drag, is commonly seen in human vehicles, as well as in birds, fish, insects and aquatic mammals. Streamlining acts to reduce the costs of transport by reducing the amount of resistance that the body experiences while moving through a fluid, by delaying separation of the flow from the surface of the object. The idea that the more streamlined the sperm head appears to be, the faster it will be able to swim is attractive, but misguided (Table 2, Figure 2). This is because sperm operate in a low Reynolds number world [14] where our intuition is often wrong. In fact, the very concept of streamlining at low Reynolds numbers is invalid, as streamline separation only occurs when inertia becomes the dominant force at work.

**Table 2:** Drag at low Reynolds numbers

Our everyday experiences of movement and of the behaviour of fluids are not necessarily applicable in situations where viscosity, not inertia, dominates. We are used to the effects of inertia, where stopping and starting require some time to occur, and where a swimming animal imparts rearward momentum to the surrounding fluid in order to move forwards. In contrast, small, slow organisms exist in a world where inertia can effectively be ignored, and viscosity dominates. This has many implications [12,68,69], but the most relevant here is that drag becomes much more important than inertia, such that when propulsion ceases, so does movement. Under such conditions, the component of drag due to the friction between the fluid and the object's surface greatly exceeds that due to pressure differences between the front and back of the object. In this case, the extra surface area realised by changing from a sphere to what we consider a 'streamlined shape', such as that of a fish, can outweigh the reduction in pressure drag.
To illustrate, figure 2 shows the relative difference in drag between a sphere and a 'streamlined' body (in this case a prolate spheroid), similar to many spermatozoan heads, at low Reynolds number. Drag with respect to volume (drag per unit volume) is likely to be most important in this context, as volume most probably determines the payload (DNA) or energy stores (mitochondria or their analogues) available to the spermatozoan, so we compare spheroids of equal volume. The conclusion is that drag on a prolate spheroid differs by maximum of 4.44% (for a 2:1 length:diameter ratio) from that of a sphere, and that for ratios higher than 4:1, drag on the 'streamlined' shape is higher than that for a sphere of equivalent volume. We suggest that it may be possible to use this relationship as a null model against which to test whether head morphology is under selection for hydrodynamic or non-hydrodynamic aspects of fertilisation success.

The peculiarities of life at low Reynolds number mean that the conventional idea of streamlining is at best futile (Table 2). The knowledge that a streamlined shape reduces drag in the Reynolds number regime of our own experience (usually *Re *>> 10,000), however, is a compelling idea and has propagated several misconceptions in the literature when applied to low Reynolds number conditions. Indeed, the processes of fluid movement are often so counter-intuitive that problems with interpretation exist throughout biology (See Vogel [12] for an accessible and engaging overview).

Although streamlining has rarely been implicated directly in determining sperm swimming velocity [but see [4,8,9]], the belief that streamlining is a 'good thing' appears to be widespread [15]. For example, Moore & Taggart [16] suggest that sperm pairing in the opossum *Monodelphis domestica*, allows the two sperm heads to form a more hydrodynamic unit than a single head. However, any reduction in drag is unlikely to offset the additional drag of a larger head unit. The combined beating and synchronisation of the two flagella [13] is, we think, a more probable explanation for the increased velocity recorded, given the energetic advantages of similar coordination in cilia [17]. In another study, Malo et al. [9] concluded that the shape of the sperm head of red deer, *Cervus elaphus hispanicus*, is a key determinant of their swimming speed. While the reasoning of these papers is essentially sound from the perspective of the human scale, the complexity of the underlying fluid dynamics means that intuition regarding movement in fluids has been misguidedly applied to the low Reynolds number regime experienced by sperm.

#### (b) The mismeasure of length

With the differences between locomotion of sperm and of larger organisms in mind, we now ask whether sperm length should influence sperm swimming velocity. There seems to be a persistent trend to cite Katz & Drobnis [18] and Gomendio & Roldan [4] as support for the idea that sperm or flagellum length is proportional to, and so determines, swimming speed. An examination of the literature provides 65 cases (plus another 5 that are ambiguous) where one or both of these papers are cited as evidence for a link between sperm or flagellum length and swimming speed. However, the points made by Katz & Drobnis [18] have frequently been misinterpreted.

Katz & Drobnis [18] discuss the forces generated by sperm movement, focusing in particular on forces generated by sperm in contact with the egg. A key statement in their conclusion is that "In general, the longer the sperm flagellum, the greater the forces generated by its motions" (p.132). Katz & Drobnis are perfectly correct in this assertion, but unfortunately it appears to have been consistently misread: the statement only deals with the forces generated by the flagellum. In the case discussed by Katz & Drobnis [18] the force generated by the flagellum is applied to an effectively stationary egg, a very different situation to the case of a free-swimming sperm.

When any object moves through a medium at a constant speed the thrust (force) required is balanced by the drag (also a force) experienced as it moves through the medium. We can simplify the case of a swimming sperm by assuming that only the flagellum produces thrust and only the head produces drag. If we assume, as do Katz & Drobnis [18], that the force (in this case thrust) produced by the flagellum is proportional to it's length (*F*_*T *_∝ *L*_*T*_), in addition we can say that the drag due to the head is proportional to some measure of it's size (in this case surface area), and the velocity at which it travels (*F*_*D *_∝ *A*_*H*_*u*). Given that surface area will be proportional to the square of some linear head measure (*A*_*H *_∝ *d*^2^), we now have a simple relationship for the balanced forces:

(1) $$L_{T}\propto d_{H}^{2}u$$

from which we can see that

(2) $$u\propto \frac{L_{T}}{d_{H}^{2}}$$

The speed attained by the sperm will therefore be proportional to the balance between drag from the head and thrust from the flagellum. We can simplify this further by noting that if *L*_*T *_>> *d*_*H*_, the ratio given above (*L*_*T*_/*d*_*H*_^2^) tends to *L*_*T*_/*d*_*H *_as L_*T *_increases. As a result we suggest that the ratio between flagellum and head length may provide a reasonable predictor for sperm swimming speed. An alternative, but more complex measure would be the ratio between flagellum length and the surface area of the head. Assuming that the head approximates a prolate spheroid, its surface area *A*_*H *_can be estimated from

(3) $$A_{H}=2\pi l_{H}^{2}+2\pi \frac{l_{H}w_{H}}{e}\sin^{-1}e$$

where *l*_*H *_is the length of the head (equatorial radius), *w*_*H *_is its width (polar radius), and *e *is the ellipticity of the spheroid, given by

(4) $$e=\sqrt{1-\frac{l_{H}^{2}}{w_{H}^{2}}}$$

The arguments above suggest that, while force generation does indeed increase with flagellum length, the implications of this for swimming speed are strongly dependent on the size (more specifically the surface area) of the head and the drag that it generates. In fact, in Table 3 and Additional file 1 we show that, because of these underlying hydrodynamic interactions, there is no reason to expect an association between sperm length and sperm velocity. Comparative studies indicate that head length tends to covary with flagellum length with either isometry or a positive allometry [3,19-21] (but see Anderson et al. [22] and Gage [23] for instances where no relationship was found). This diversity of patterns in head to flagellum scaling in different groups can explain the diversity of total length to velocity relationships found in the literature (Table 3).

**Table 3:** Heads or tails?

The simplified argument that velocity is not likely to be determined purely by length alone (equation (2)), is supported by the results from both slender body theory [70-74], and the simpler, but less accurate, resistive force theory [both reviewed by [14]]. Both treatments indicate that drag due to the head, and the hydrodynamic interaction between the head and the flagellum of a sperm, will both play a role in determining forward speed.

As few studies consider multiple length measures as well as speed [e.g. [9]], and none provide adequate data for further analysis, we used a reanalysis of Higdon's [71] results (see Additional file 1) to estimate relative forward swimming speeds for sperm of a range of different species whose head and flagellum lengths were given in the literature. Where raw data were unavailable, data points were extracted from published figures using GraphClick^® ^(Arizona software, http://www.arizona-software.ch). Our flagellum length measures represent the flagellum plus midpiece, except for fishes where the midpiece is an integral part of the head. All analyses were carried out using R v. 2.5.1 [75] with the SMATR package [76]. No phylogenetic correction was used, as the necessary data were not available, and because we were interested in the patterns resulting from different scaling relationships, not the form of individual relationships *per se*. Reduced Major Axis (RMA) regression was used to describe the relationship between sperm head length and flagellum length (Additional file 1). We decided between linear and power functions (log head vs. log flagellum length) on the basis of the amount of variation explained by the two models, selecting the one with the higher *R*^2^. We arbitrarily designated an adjusted *R*^2 ^of 15% as the cut-off for the percentage variance explained by the regression model before we considered there to be no relationship between the two variables. We characterised data where no relationship was found by the ratio of variances between them, and represent these cases by vertical and horizontal lines in the figures.

**Figure 1:**
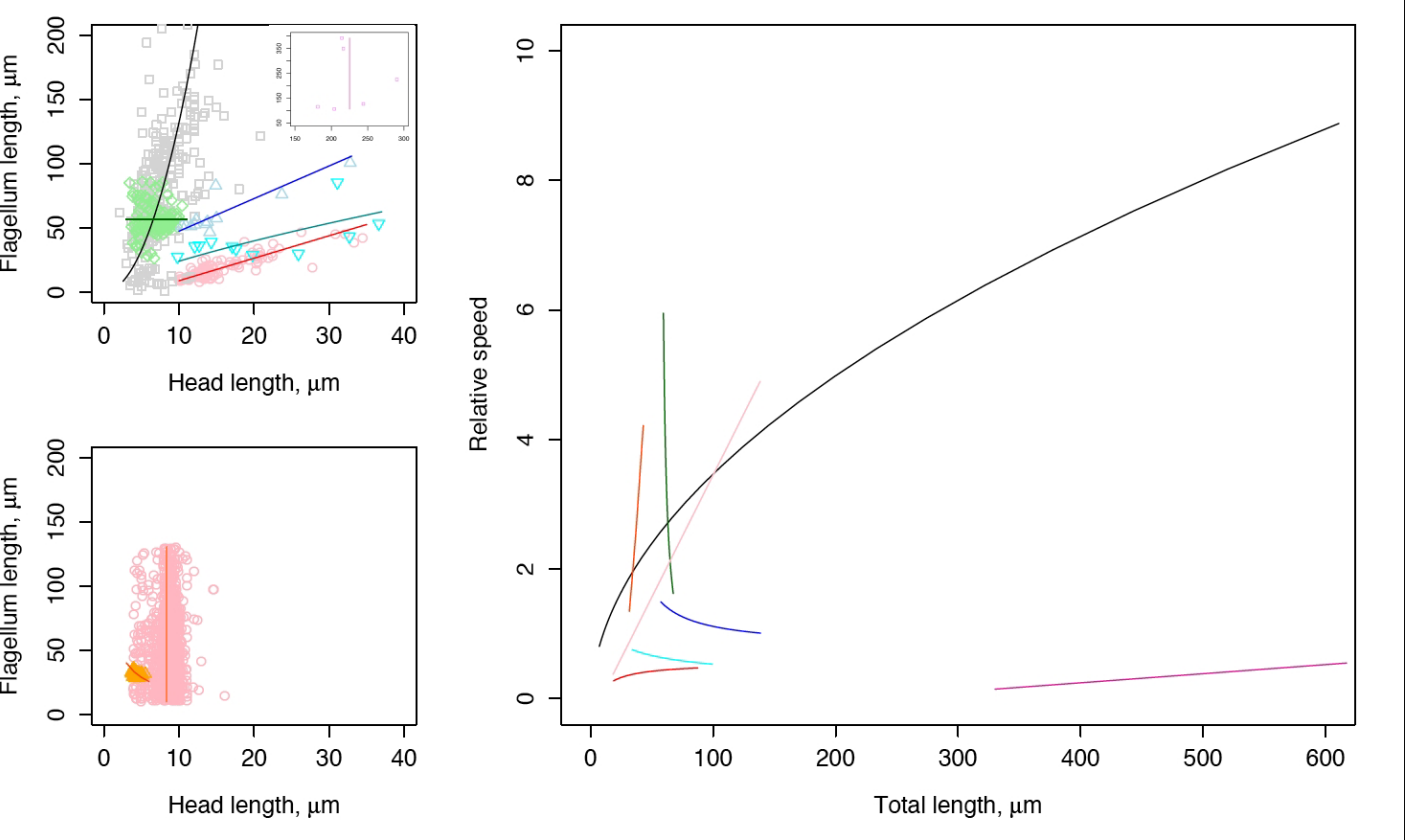
**Recorded relationships between head length and flagellum length**. Upper left panel *interspecific *studies: Black squares – mammals ; Red circles – frogs ; Blue up triangles – shorebirds ; Green diamonds – mammals ; Cyan down triangles – frogs. Inset: Violet squares – beetles. Lower left panel *intraspecific *studies: Pink circles - boar ; Orange up triangles – salmon. Solid lines indicate RMA regression lines, vertical and horizontal lines are non-significant relationships. Right hand panel: *Resulting relationships between total sperm length and predicted speed*. Colours correspond to the studies in the left had panels. Note the range of possible patterns, dependent on the scaling parameter *c *: Black – positive (*c *= 1.0); Red – positive (*c *= 1.0); Blue – negative (c = 1.0); Green – negative (*b *= 0.0); Cyan – negative (*c *= 0.46); Violet – positive (*b *= 8); Pink – positive (*b *= 8); and Orange – positive (*c *= -0.29). Citations for the studies used are given in the Additional file 1.

Figure 1 illustrates that the allometry of head and flagellum lengths appears to be taxon-specific and not consistent across species. We next plotted total sperm length (head plus flagellum) against our estimates of swimming speed (Figure 1, rhs). The result is a mix of patterns that cannot be predicted from knowledge of total length alone. Qualitatively similar patterns are seen when other single length measures, such as flagellum length, are used to estimate speed instead of total length. The diversity of patterns also remains if the linear relationship between head and flagellum is relaxed to include curvilinear relationships (data not shown).

These results show that the sperm length-velocity relationships commonly reported to take a number of forms (including no apparent link) can likely be explained by the scaling between structural components of sperm cells. It is impossible to consistently predict sperm swimming speed from knowledge of length parameter alone, so it is not surprising that previous studies attempting to link the two have been unsuccessful. However, use of the ratio of head to flagellum length can provide insight into swimming velocity.

We focus on sperm length to illustrate that the simple measures used in the majority of sperm competition studies are inadequate to allow proper understanding of the link between sperm morphology and swimming speed. However, flagellar beat dynamics are a primary determinant of swimming speed [14,71,73,77] with swimming velocity highly dependent on the beat amplitude of the flagella. Thus future studies should attempt to characterise sperm kinematics if we are to fully understand the link between morphology and velocity of sperm.

A corollary of the above arguments is that several relationships between flagellum and head lengths result in a total length-to-velocity relationship that is an asymptotic function of total length (figure 1). This has an important implication, in that if total length is selected upon by the female reproductive tract [as suggested by [24]] increasing sperm length (past a critical threshold for negative slopes, or for all cases for positive slopes) is not detrimental to the sperm's swimming speed. This suggests that past a critical total length we can expect selection for speed to be weakened such that further elongation of the flagellum with respect to a given head size does not change the speed of the sperm. In these instances, selection on flagellum or total length, for example via cryptic female choice (see below), can act to influence sperm length without detrimental effects on swimming speed.

## Discussion

We suggest that, irrespective of which measure (flagellum length, total length, or head length) is used, attempts to correlate any single measure of length to speed are likely to be futile. In fact, we argue that the rather confusing patterns reported in the literature are due to the use of single measures of sperm length, and that accounting for the balance between drag from the head and thrust from the flagellum will allow us to extend our understanding of the link between sperm form and function.

In this context, we start our discussion by reviewing the literature on sperm length in order to address three key questions relating to the assumption that longer sperm swim faster and are more competitive:

### (a) Does sperm competition favour increased sperm length?

A number of studies suggest that the selective pressure of sperm competition extends beyond simply favouring males producing larger ejaculates [reviewed by [2]]. For example, it is commonly argued that longer sperm will be favoured by selection for increased swimming performance, and therefore enhanced competitiveness [4,8]. Other theories include links between sperm length and longevity [1], between midpiece size and energy availability [25], and between size and ability to displace smaller sperm from the female tract [26]. These ideas have gained weight in the light of several comparative studies that show that average sperm lengths are greater in species where polyandry (and therefore sperm competition) is more prevalent [3-5,21,27-30]. However, this pattern is not universal and the magnitude of sperm competition can be negatively associated with sperm length [31,32], while in other groups there is no significant association between indices of sperm competition (e.g. testes size, social group size) and sperm length (e.g. bats: [33], 31 spp. of primates: [34], 83 spp. of mammals: [35]). Across species of passerine birds, positive-, negative- and no- relationship(s) have all been found between levels of sperm competition and sperm midpiece length [20], while in primates the volume of the midpiece is greater in species with relatively large testes and polyandrous mating systems [34]. Snook [2] summarizes most of these studies in her Table 1. In short, the comparative data clearly fail to show a consistent relationship between the risk (probability of ejaculates of two males competing for fertilization) or intensity (the number of ejaculates competing for fertilization) of sperm competition and sperm length.

### (b) Does sperm length enhance competitive success?

Few studies have tested specifically whether variation in relative sperm length among rival males influences competitive fertilization success, and thus whether intrasexual selection has the potential to act on this trait. Two studies have reported a fertilization advantage for relatively larger sperm in species with aflagellate amoeboid sperm [26,36]. However, we are here concerned with the structure and function of flagellate sperm. In the snail *Viviparus ater *the length of oligopyrene sperm was the best predictor of relative paternity, explaining 38% of the deviance in second male paternity in competitive fertilization trials [37]. However, oligopyrene sperm do not contain the full complement of chromosomes and do not fertilize eggs. While the function of oligopyrene sperm remains unknown, it is clear that these data cannot be taken as evidence for a functional relationship between the length and performance of fertilizing sperm. While Gage et al. [38] failed to find a relationship between total sperm length and competitive fertilization success in Atlantic salmon *Salmo salar*, Vladiæ et al. [7] reported that in this species the length of the flagellum's end piece did predict fertilization ability. By contrast, Gage & Morrow [39] reported that crickets with relatively shorter sperm have a fertilization advantage, while others report no evidence for a role of sperm length in influencing competitive fertilization success in crickets [40,41], or bluegill sunfish [42].

### (c) Does sperm length predict swimming speed?

Empirical evidence that sperm swimming speed is a positive function of a measure of sperm length is limited (Table 1). A positive relationship between total sperm length and maximum sperm swimming velocity was reported in a comparative study across five species of mammal [4], with recent reanalysis and control for phylogeny at the family level providing further support [8]. In an intraspecific study of red deer, Malo et al. [9] found that sperm with *shorter *midpieces but longer heads and longer relative components of the flagella swam more quickly than sperm with long midpieces, shorter heads and relatively shorter flagella components. While Cardullo & Baltz [25] postulate a link between energy supply, speed and the determination of flagellum length by mitochondrial volume. Pitcher et al [10] reported a positive relationship between head length and speed in the guppy, *Poecilia reticulata*, but not between other length measures. Elsewhere, others have failed to establish a relationship between sperm swimming speeds and any component of sperm length [e.g. [42-47]]. For example, Minoretti and Baur [47] found no relationship between total sperm length and sperm swimming velocity in the hermaphroditic land snail *Arianta arbustorum*. Likewise, Birkhead et al.'s [43] study of zebra finches *Taeniopygia guttata *revealed no evidence that the length of the sperm flagellum, midpiece or tail were correlated with sperm swimming speed, while Gage et al. [23,46] similarly found no relationship between total sperm or flagella length and sperm motility in Atlantic salmon *Salmo salar*. The assertion that longer sperm exhibit faster swimming velocities is therefore largely unsupported by the available evidence.

### (d) Female-derived effects on sperm evolution

**Figure 3:**
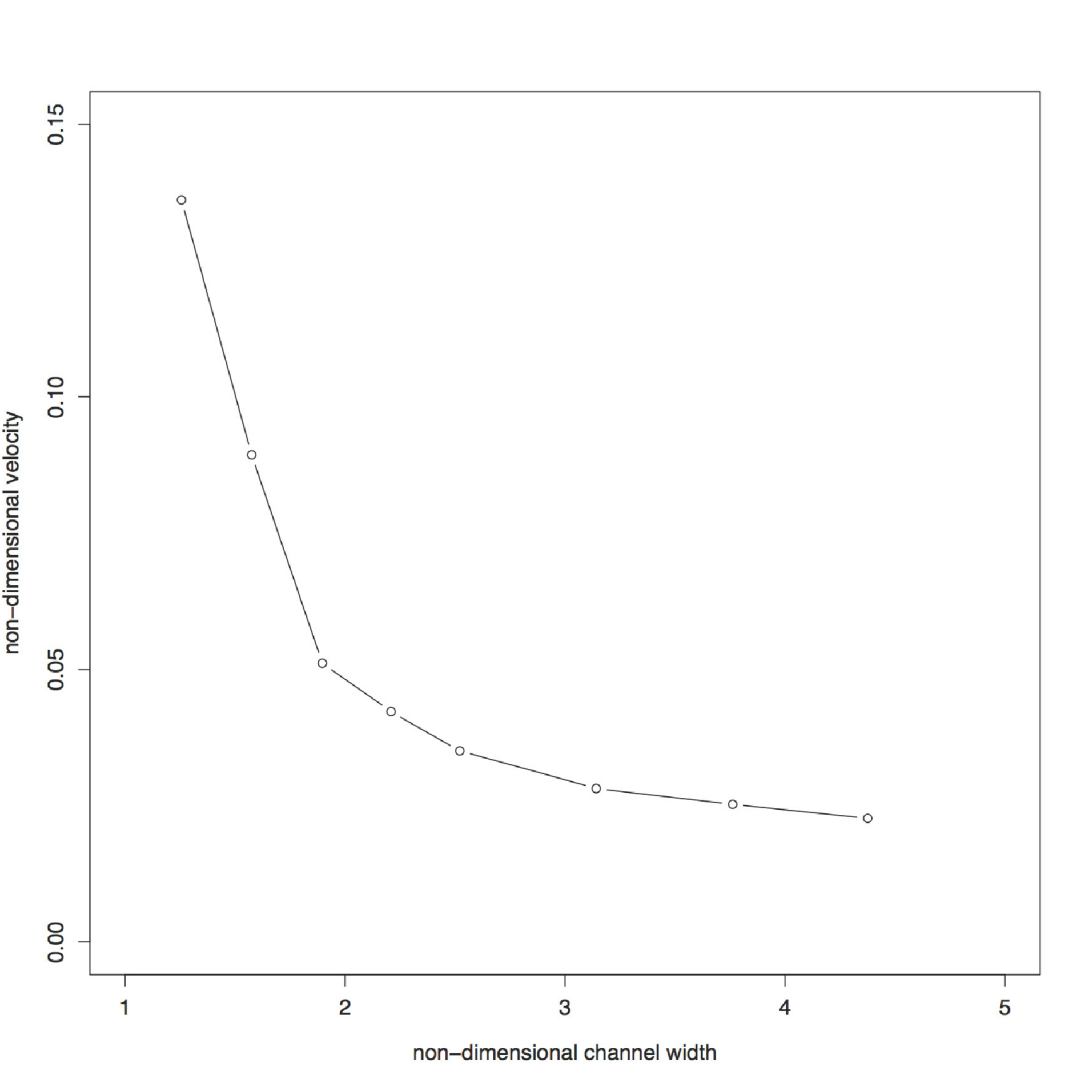
**Computed sperm velocity as a function of channel width**. Swimming speed is predicted to increase dramatically as the channel walls become closer. Non-dimensionalised terms are velocity/wave speed of the flagellum and channel width/amplitude of flagellar beat. Redrawn from [81].

Finally, while the simplicity of relating morphology to velocity in sperm is attractive, a further source of selection on sperm phenotype comes from cryptic female choice [48-51]. For example, in internally fertilizing species, sperm must operate inside the females' reproductive tract. Due to conditions that engineers call 'wall effects', the lining of the female reproductive tract can have important effects on sperm performance, because the movement of fluid generated by a swimming sperm becomes constrained when it approaches a solid boundary (Table 4 and Figure 3). This 'wall effect' can take several forms, the two most commonly studied of which have been changes in swimming speed, and attraction of the sperm to the wall surface. All sperm encounter solid surfaces in the form of the egg, but in internally fertilizing species, the female reproductive tract means that a solid (but flexible) surface is present for the entire functional lifetime of the sperm. In addition to influencing movement of sperm in internal fertilizers, and providing a potential mechanism allowing females to manipulate sperm in vivo, the unfortunate combination of speed changes due to the presence of boundaries, and attraction to those boundaries, poses a potential problem for current sperm velocity measurement techniques in vitro, and the biological significance of those patterns. Velocity measurements from samples on slides may not be truly comparable between studies if the impact of wall effects is not quantified, and the swimming velocities achieved by sperm on slides may not represent those achieved in their natural environments.

**Table 4:** Direct effects of surfaces on sperm performance

Vogel [12] gives a particularly disquieting illustration of the problem wall effects may present in studies of microscopic movement: at a Reynolds number of 10^-3 ^(slightly less than that of a sea urchin spermatozoon), a wall 50 diameters away can significantly influence drag of a cylinder moving parallel to the wall. Changes in swimming speed due to wall effects are predicted to be modulated by drag effects, but as often with fluid dynamics, this effect is not always intuitive: sperm are predicted to swim *faster *within 10 body lengths of a wall, than in an unbounded fluid [78,79]. Using sea urchin (*Arbacia punctulata*) sperm Gee & Zimmer-Faust [80] found significant differences in speed between sperm swimming at two different distances from a wall and concluded that wall effects can "substantially exaggerate swimming speed" (p 3185). This finding is supported by the theoretical predictions of [81] (Figure 3). Vogel [12] suggests a rule of thumb derived from White [82], that for *Re *< 1, we can be reasonably sure that wall effects can be ignored if
(5) yL>20Re⁡
where *y *is the distance to the nearest wall, and *L *is the characteristic length of the object (in this case total sperm length).
The attraction of sperm to walls, such as glass coverslips, and cell surfaces (such as that of the egg) seems to have been first noted by Dewitz (1886) and quantified initially by Rothschild (1963). Since then several empirical and theoretical studies have been conducted on this phenomenon. Winet et al [83] used human sperm to study accumulation at boundaries, while Woolley [84] used a selection of sperm from mice, chinchillas, *Xenopus *and eels, and Cosson et al. [85] worked with sea urchin sperm.
Fauci & MacDonald [81] used a numerical approach to further explore the effects of boundaries on sperm motion, concluding that hydrodynamic effects lead to the attraction of sperm to boundaries. However, the exact mechanisms involved may depend on the type of swimming motion [84] or asymmetries in the head-flagellum connection [85].

The potential importance of female-derived effects can be seen in the increasing body of comparative work on mammals [52,53], birds [54,55], and insects [5,56-61] showing that sperm length is positively associated with the length of the sperm storage organs of females. Interestingly, closer examination of the relationship between sperm competition and sperm length in birds reveals that it is selection from the female reproductive tract that directly explains variation in sperm length [62]. Thus, sperm length appears to respond positively to evolutionary increases in female reproductive tract dimensions [52]. Female sperm choice, mediated by active transport of sperm by the female coupled with variation in female reproductive tract morphology, therefore seems a highly plausible hypothesis to help explain sperm-female coevolution.

We also note that there is no *a priori *reason to expect cryptic female choice to favour long or short sperm. Indeed, female choice often imposes divergent patterns of selection across taxonomic groups so that positive or negative associations between sperm length and the degree of polyandry are equally accommodated by the cryptic female choice hypothesis. García-González and Simmons [63] have recently shown that in dung beetles (*Onthophagus taurus*) males with relatively short sperm enjoy higher reproductive success than their longer-sperm rivals during competitive fertilization trials. However, in their study they also showed that the success of males with different sperm lengths was contingent on the size of the female's sperm storage organs: the advantage of shorter sperm was greater when competing for fertilization in females with larger spermathecae [63]. Crucially, a recent quantitative genetic analysis of male and female reproductive traits confirmed that sperm length is genetically correlated (negatively) with spermathecal size [64], providing explicit support for the evolution of sperm phenotypes via cryptic female choice.

The results from dung beetles parallel previous findings in *Drosophila melanogaster*, where sperm length likewise interacts with female reproductive tract morphology to determine the reproductive success of competing ejaculates [24,65]. In the case of *Drosophila*, however, males with longer sperm have the fertilization advantage, illustrating the divergent nature of cryptic female choice acting on sperm morphology. Again, these studies also provided evidence that increased sperm length was genetically correlated with female reproductive tract morphology: selection for increased seminal receptacle length in female *D. melanogaster *generated a correlated response in sperm length, a result expected if these traits are in linkage disequilibrium [24].

## Conclusion

It seems clear that some assumptions regarding the physics of sperm locomotion have hampered our progress in understanding the processes mediating sperm competition. This synthesis of literature from diverse fields highlights the problems of thinking about biological evolution in isolation from physical constraints. While relating sperm length to speed has clear implications for sperm competition, there is a lack of consistent evidence linking the two. However, by taking account of fluid dynamic interactions it is possible to reconcile these conflicting patterns, which should allow future work in this area to progress rapidly. Scaling of the flagellum:head ratio may be more important than simplistic ideas of large and small sperm with differing morphologies. However, due to the influence of wall effects, sperm competition is unlikely to act on sperm length and motility in isolation from female effects. This suggests to us that definitive links between and measure(s) of sperm length are unlikely to be found consistently in internally fertilising species, further complicating any attempts to correlate length to swimming speed. Nonetheless, incorporating realistic fluid dynamics into our theories should enable progress in this area.

We recommend that future studies examine the flagellum:head ratio as a potential link between sperm form and function, but recognise that its use is particularly appropriate for external fertilisers, as this group will be least affected by wall effects, and has almost instantaneous fertilisation so that sperm longevity can be ignored. While external fertilisers may offer the best opportunity to test our ideas, internal fertilisers will also be affected in the same way, albeit with the additional effects of walls and other mechanisms of female cryptic choice. We also advocate the reporting of slide-well depths and sperm diameters in all studies and encourage the development of velocity measurement methods less influenced by wall effects than current techniques.

We suggest that physical constraints on sperm swimming ability, coupled with the potential for sperm-female coevolution, demand a critical reappraisal of the belief that sperm competition selects for longer, more powerful sperm. Taking account of physics in this way may lead to the development of a framework that allows for non-mutually exclusive alternative explanations for why sperm phenotype seems to exhibit such great variation.

## Authors' contributions

SH carried out the mathematical analysis. All authors jointly conceived of the study, helped to draft, and approved, the final manuscript.

## Supplementary Material

Additional File 1 supplementary materials. Click here for file

## References

[B1] Parker GA Sperm competition games – sperm size and sperm number under adult control Proc Biol Sci 1993 253 1338 245 254 8234363 10.1098/rspb.1993.0110

[B2] Snook RR Sperm in competition: not playing by the numbers Trends Ecol Evol 2005 20 1 46 53 16701340 10.1016/j.tree.2004.10.011

[B3] Byrne PG Simmons LW Roberts JD Sperm competition and the evolution of gamete morphology in frogs Proc Biol Sci 2003 270 1528 2079 2086 14561298 10.1098/rspb.2003.2433 PMC1691467

[B4] Gomendio M Roldan ERS Sperm competition influences sperm size in mammals Proc Biol Sci 1991 243 1308 181 185 1675796 10.1098/rspb.1991.0029

[B5] Morrow EH Gage MJG The evolution of sperm length in moths Proc Biol Sci 2000 267 1440 307 313 10714886 10.1098/rspb.2000.1001 PMC1690520

[B6] Parker G Sperm competition and its evolutionary consequences in the insects Biol Rev 1970 45 525 567

[B7] Vladić TV Afzelius BA Bronnikov GE Sperm quality as reflected through morphology in salmon alternative life histories Biol Reprod 2002 66 98 105 11751270 10.1095/biolreprod66.1.98

[B8] Gomendio M Roldan E Implications of diversity in sperm size and function for sperm competition and fertility Int J Dev Biol 2008 52 5–6 439 447 18649256 10.1387/ijdb.082595mg

[B9] Malo AF Gomendio M Garde J Lang-Lenton B Soler AJ Roldan ERS Sperm design and sperm function Biol Letts 2006 2 246 249 17148374 10.1098/rsbl.2006.0449 PMC1618917

[B10] Pitcher T Rodd F Rowe L Sexual colouration and sperm traits in guppies J Fish Biol 2007 70 165 177

[B11] Cohen J Reproduction 1977 London: Butterworths

[B12] Vogel S Life in moving fluids 1994 Princeton: Princeton University Press

[B13] Taylor GI Analysis of the swimming of microscopic organisms Proc Roy Soc Lond A 1951 209 447 461

[B14] Brennen C Winet H Fluid mechanics of propulsion by cilia and flagella Ann Rev Fluid Mech 1977 9 339 398

[B15] Ainsworth C The secret life of sperm Nature 2005 436 770 771 16094338 10.1038/436770a

[B16] Moore HDM Taggart DA Sperm pairing in the Oppossum increases the efficiency of sperm movement in a viscous environment Biol Reprod 1995 52 947 953 7780016 10.1095/biolreprod52.4.947

[B17] Gueron S Levit-Gurevich K Energetic considerations of ciliary beating and the advantage of metachronal coordination Proc Nat Acad Sci 1999 96 12240 12245 10535905 10.1073/pnas.96.22.12240 PMC22900

[B18] Katz DF Drobnis EZ Bavister BD, Cummins J, Roldan ERS Analysis and interpretation of the forces generated by spermatozoa Fertilization in mammals 1990 Serono Symposia 125 137

[B19] Gage MJG Mammalian sperm morphometry Proc Roy Soc Lond B 1998 265 97 103 10.1098/rspb.1998.0269 PMC1688860 9474794

[B20] Immler S Birkhead TR Sperm competition and sperm midpiece size: no consistent pattern in passerine birds Proc Biol Sci 2007 274 1609 561 568 17476777 10.1098/rspb.2006.3752 PMC1766377

[B21] Johnson DDP Briskie JV Sperm competition and sperm length in shorebirds Condor 1999 101 4 848 854

[B22] Anderson MJ Nyholt J Dixson AF Sperm competition and the evolution of sperm midpiece volume in mammals J Zool 2005 267 135 142

[B23] Gage MJG Stockley P Parker GA Sperm morphometry in the Atlantic salmon J Fish Biol 1998 53 835 840

[B24] Miller GT Pitnick S Sperm-female coevolution in *Drosophila* Science 2002 298 1230 1233 12424377 10.1126/science.1076968

[B25] Cardullo RA Baltz JM Metabolic regulation in mammalian sperm: mitochondrial volume determines sperm length and flagellar beat frequency Cell Motil Cytoskeleton 1991 19 3 180 188 1878988 10.1002/cm.970190306

[B26] LaMunyon CW Ward S Larger sperm outcompete smaller sperm in the nematode *Caenorhabdites elegans* Proc Biol Sci 1998 265 1409 1997 2002 9821364 10.1098/rspb.1998.0531 PMC1689481

[B27] Gage MJG Associations between body size, mating pattern, testis size and sperm lengths across butterflies Proc Roy Soc Lond B 1994 258 247 254

[B28] Baer B Schmid-Hempel P Hoeg JT Boomsma JJ Sperm length, sperm storage and mating system characteristics in bumblebees Insectes Sociaux 2003 50 101 108

[B29] Calhim S Immler S Birkhead TR Postcopulatory sexual selection is associated with reduced variation in sperm morphology PLoS ONE 2007 2 5 e413 17476335 10.1371/journal.pone.0000413 PMC1855076

[B30] Balshine S Leach BJ Neat F Werner NY Montgomerie R Sperm size of African cichlids in relation to sperm competition Behav Ecol 2001 12 6 726 731

[B31] Schulte-Hostedde AI Millar J Intraspecific variation of testis size and sperm length in the yellow-pine chipmunk (*Tamias amoenus*): implications for sperm competition and reproductive success Behav Ecol Sociobiol 2004 55 272 277

[B32] Stockley P Gage MJG Parker GA Møller AP Sperm competition in fishes: the evolution of testis size and ejaculate characteristics Am Nat 1997 149 5 933 954 18811256 10.1086/286031

[B33] Hosken DJ Sperm competition in bats Proceedings of the Royal Society London B 1997 264 385 392 10.1098/rspb.1997.0055 PMC1688272 9107054

[B34] Anderson MJ Dixson AF Motility and the mipiece in primates Nature 2002 416 496 11932733 10.1038/416496a

[B35] Gage MJG Freckleton RP Relative testis size and sperm morphometry across mammals: no evidence for an association between sperm competition and sperm length Proc Biol Sci 2003 270 1515 625 632 12769463 10.1098/rspb.2002.2258 PMC1691278

[B36] Radwan J Interspecific variation in sperm competition success in the bulb mite: a role for sperm size Proc Roy Soc Lond B 1996 263 855 859

[B37] Oppliger A Naciri-Graven Y Ribi G Hosken DJ Sperm length influences fertilization success during sperm competition in the snail *Viviparus ater* Mol Ecol 2003 12 2 485 492 12535098 10.1046/j.1365-294x.2003.01748.x

[B38] Gage MJG Macfarlane CP Yeates S Ward RG Searle JB Parker GA Spermatozoal traits and sperm competition in Atlantic salmon: relative sperm velocity is the primary determinant of fertilisation success Curr Biol 2004 14 44 47 14711413

[B39] Gage MJG Morrow EH Experimental evidence for the evolution of numerous, tiny sperm via sperm competition Curr Biol 2003 13 9 754 757 12725733 10.1016/s0960-9822(03)00282-3

[B40] Morrow EH Gage MJG Sperm competition experiments between lines of crickets producing different sperm lengths Proc Biol Sci 2001 268 1482 2281 2286 11674877 10.1098/rspb.2001.1807 PMC1088877

[B41] Simmons LW Wernham J Garcia-Gonzalez F Kamien D Variation in paternity in the field cricket *Teleogryllus oceanicus*: no detectable influence of sperm numbers or sperm length Behav Ecol 2003 14 4 539 545

[B42] Stoltz JA Neff BD Sperm competition in a fish with external fertilization: the contribution of sperm number, speed and length J Evol Biol 2006 19 6 1873 1881 17040384 10.1111/j.1420-9101.2006.01165.x

[B43] Birkhead TR Pellatt EJ Brekke P Yeates R Castillo-Juarez H Genetic effects on sperm design in the zebra finch Nature 2005 434 7031 383 387 15772662 10.1038/nature03374

[B44] Burness G Casselman SJ Schulte-Hostedde AI Moyes CD Montgomerie R Sperm swimming speed and energetics vary with sperm competition risk in bluegill (*Lepomis macrochirus*) Behav Ecol Sociobiol 2004 56 1 65 70

[B45] Fitzpatrick JL Desjardins JK Milligan N Montgomerie R Balshine S Reproductive-tactic-specific variation in sperm swimming speeds in a shell-brooding cichlid Biol Reprod 2007 77 2 280 284 17460159 10.1095/biolreprod.106.059550

[B46] Gage MJG Macfarlane CP Yeates S Shackleton R Parker GA Relationships between sperm morphometry and sperm motility in the Atlantic salmon J Fish Biol 2002 61 1528 1539

[B47] Minoretti N Baur B Among- and within-population variation in sperm quality in the simultaneously hermaphroditic land snail *Arianta arbustorum* Behav Ecol Sociobiol 2006 60 270 280

[B48] Eberhard WG Female Control: Sexual Selection by Cryptic Female Choice 1996 Princeton: Princeton University Press

[B49] Keller L Reeve H Why do females mate with multiple males? The sexually selected sperm hypothesis Adv Stud Behav 1995 24 291 315

[B50] Thornhill R Cryptic female choice and its implications in the scorpionfly *Harpobittacus nigricepts* Am Nat 1983 122 765 788

[B51] Birkhead TR Cryptic female choice: criteria for establishing female sperm choice Evolution 1998 52 1212 1218 10.1111/j.1558-5646.1998.tb01848.x 28565225

[B52] Briskie JV Montgomerie R Sperm size and sperm competition in birds Proc Roy Soc Lond B 1992 247 1319 89 95 10.1098/rspb.1992.0013 1349186

[B53] Anderson MJ Dixson AS Dixson AF Mammalian sperm and oviducts are sexually selected: evidence for co-evolution J Zool 2006 270 4 682 686

[B54] Briskie JV Montgomerie R Patterns of sperm storage in relation to sperm competition in passerine birds Condor 1993 95 2 442 454

[B55] Birkhead TR Briskie JV Moller AP Male sperm reserves and copulation frequency in birds Behav Ecol Sociobiol 1993 32 2 85 93

[B56] Minder AM Hosken DJ Ward PI Co-evolution of male and female reproductive characters across the Scathophagidae (Diptera) J Evol Biol 2005 18 1 60 69 15669961 10.1111/j.1420-9101.2004.00799.x

[B57] Sasakawa K Sperm bundle and reproductive organs of carabid beetles tribe Pterostichini (Coleoptera: Carabidae) Naturwissenschaften 2007 94 5 384 391 17165077 10.1007/s00114-006-0200-4

[B58] Presgraves DC Baker RH Wilkinson GS Coevolution of sperm and female reproductive tract morphology in stalk-eyed flies Proc Roy Soc Lond B 1999 266 1423 1041 1047

[B59] Dybas LK Dybas HS Coadaptation and taxonomic differentiation of sperm and sperathecae in featherwing beetles Evolution 1981 35 1 168 174 10.1111/j.1558-5646.1981.tb04869.x 28563443

[B60] Pitnick S Markow T Spicer G Delayed male maturity is a cost of producing large sperm in Proc Natl Acad Sci U S A 1995 92 23 10614 10618 7479851 10.1073/pnas.92.23.10614 PMC40662

[B61] Pitnick S Markow TA Spicer GS Evolution of multiple kinds of female sperm-storage organs in *Drosophila* Evolution 1999 53 6 1804 1822 10.1111/j.1558-5646.1999.tb04564.x 28565462

[B62] Briskie JV Montgomerie R Birkhead TR The evolution of sperm size in birds Evolution 1997 51 3 937 945 10.1111/j.1558-5646.1997.tb03674.x 28568571

[B63] García-González F Simmons LW Shorter sperm confer higher competitive fertilization success Evolution 2007 61 4 816 824 17439614 10.1111/j.1558-5646.2007.00084.x

[B64] Simmons L Kotiaho J Quantitative genetic correlation between trait and preference supports a sexually selected sperm process Proc Nat Acad Sci 2007 104 42 16604 16608 17921254 10.1073/pnas.0704871104 PMC2034270

[B65] Pattarini JA Starmer WT Bjork A Pitnick S Mechanisms underlying the sperm quality advantage in *Drosophila melanogaster* Evolution 2006 60 10 2064 2080 17133863

[B66] Leach B Montgomerie R Sperm characteristics associated with different male reproductive tactics in bluegills (*Leopmis macrochirus*) Behav Ecol Sociobiol 2000 49 31 37

[B67] Locatello L Pilastro A Deana R Zarpellon A Rasotto M Variation pattern of sperm quality traits in two gobies with alternative mating tactics Func Ecol 2007 21 975 981

[B68] Berg HC Random walks in biology 1983 Princeton, NJ: Princeton University Press

[B69] Purcell EM Life at low Reynolds numbers Am J Physiol 1977 45 3 11

[B70] Hancock GJ The self-propulsion of microscopic organisms through liquids Proc Roy Soc Lond A 1953 217 1182 96 121

[B71] Higdon JJL A hydrodynamic analysis of flagellar propulsion J Fluid Mech 1979 90 4 685 711

[B72] Johnson RE Brokaw CJ Flagellar hydrodynamics – a comparison between resistive-force theory and slender-body theory Biophys J 1979 25 113 127 262381 10.1016/S0006-3495(79)85281-9 PMC1328451

[B73] Higdon JJL The hydrodynamics of flagellar propulsion: helical waves J Fluid Mech 1979 94 2 331 351

[B74] Dresdner RD Katz DF Berger SA The propulsion by large amplitude waves of uniflagellar micro-organisms of finite length J Fluid Mech 1980 97 3 591 621

[B75] R Development Core Team R: A language and environment for statistical computing 2006

[B76] Warton DI Ormerod J SMATR: (Standardised) Major Axis Estimation and Testing Routines R package version 2.0 2005

[B77] Dillon RH Fauci LJ Omoto C Yang X Fluid dynamic models of flagellar and ciliary beating Ann NY Acad Sci 2007 1101 494 505 17344534 10.1196/annals.1389.016

[B78] Katz DF On the propulation of micro-organisms near solid boundaries J Fluid Mech 1974 64 33 49

[B79] Katz DF Blake JR Paveri-Fontana SL On the movement of slender bodies near plane boundaries at low Reynolds number J Fluid Mech 1975 72 529 540

[B80] Gee CC Zimmer-Faust RK The effects of walls, paternity and ageing on sperm motility J Exp Biol 1997 200 3185 3192 9364024 10.1242/jeb.200.24.3185

[B81] Fauci LJ McDonald A Sperm motility in the presence of boundaries Bull Math Biol 1995 57 5 679 699 7606221 10.1007/BF02461846

[B82] White C The drag of cylinders in fluids at slow speeds Proc Roy Soc Lond A 1946 186 472 479

[B83] Winet H Bernstein GS Head J Observations on the response of human spermatozoa to gravity, boundaries and fluid shear J Reprod Fertil 1984 70 511 523 6699814 10.1530/jrf.0.0700511

[B84] Woolley DM Motility of spermatozoa at surfaces Reproduction 2003 126 2 259 270 12887282 10.1530/rep.0.1260259

[B85] Cosson J Huitorel P Gagnon C How spermatozoa come to be confined to surfaces Cell Motil Cytoskeleton 2003 54 1 56 63 12451595 10.1002/cm.10085

