# Supplementary material for: Sperm competition: linking form to function
Source: BMC Evol Biol. 2008 Nov 25;8:319. doi: 10.1186/1471-2148-8-319 (PMC2632676; doi:10.1186/1471-2148-8-319)
Supplement: Additional File 1 — supplementary materials. [file 1471-2148-8-319-S1.pdf]

Supplementary Material

The linear relationship between forward speed *U* and the ratio of flagellum (*L*) to head (*d*) length allows us to provide a simple rule for estimating the form of the *L/d to* forward velocity relationship.

The ratio *L/d* can also be extracted (SH *pers obs*) from the resistive-force theories outlined in Brennen & Winet's [1] seminal review, and by the use of energy-balance equations illustrated by Denny [2]. One of the most accurate mathematical descriptions of sperm movement to date comes from Higdon [3, 4]. Again, although not explicit in his work, Higdon's [3, 4] results can be reinterpreted in the context of sperm competition. For the simple sperm Higdon considered (a spherical head with a flagellum), we are able to use his results to show that, as expected, there is a near-linear relationship between flagellum length and forward velocity *for a given head radius*. To allow for sperm with non-spherical heads, we use head length rather than radius. We denote head length as the longest axis of the head, which we assume to be parallel to the axis of travel. **Figure 1** illustrates that there is a nonlinear relationship between beat amplitude and velocity, and the importance of the flagellum/head ratio.

Assuming that (1) the midpiece can be treated as part of one or other of the head or flagellum, and (2) that head and flagellum dimensions other than length scale isometrically, then given that

$$U \propto \frac{L}{d},$$

(A1)

and if flagellum length scales as

$$L = a + b \cdot d^c.$$

(A2)

where *a*, *b* and *c* are scaling constants. Then

$$\frac{L}{d} = \frac{a}{d} + b \cdot d^{c-1}$$

(A3)

and so

$$U \propto \frac{a}{d} + b \cdot d^{c-1}.$$

(A4)

When we consider total sperm length L_t the situation becomes considerably more complex as we must deal with the additive effects of two terms (*L* and *d*), which are correlated via the general relationship defined in eqn (A2).

For the special case where *L* is a linear function of *d* alone (i.e *a* = 0 and *c* = 1 so that *L* scales isometrically with *d*), equation (A4) reduces to

$$U \propto \frac{0}{d} + b \cdot d^0 \propto b,$$

(A5)

indicating that when the ratio L/d is constant, so is speed. That is, speed does not change with increasing tail length, if (and only if) head length increases at exactly the same rate.

In order to examine the relationship between L_t and U we first find L_t as a function of d:

$$L_t = L + d = a + b \cdot d^c + d$$

(A6)

In the special case of isometry between L and d this reduces to

$$L_t = b \cdot d + d \, .$$

(A7)

By substituting eqn (A5) into this expression we find that

$$L_t \propto d(U+1)$$

(A8)

so that now we can redefine U in terms of L_t

$$U \propto \frac{L_t}{d} - 1$$

(A9)

When L_t is plotted against U, equation (A9) gives a positive correlation between L_t and U, with a slope that becomes shallower as $d \to \infty$. Thus for isometrically scaling head and flagellum lengths, total sperm length will correlate positively with speed. However, as we show here, because the relationship between L_t and U depends on the way in which d and L scale, many different patterns for U as a function of L_t can be found.

Unfortunately, the general form of eqn (A6), and subsequently the equivalent of eqn (A9), is difficult to solve, but below we give some examples of closed-form solutions for different values of *c*. However, if the relationship between *d* and *L* is known, we can simply plot L_t, the sum of *d* and *L*, against a relative measure of *U* (the ratio *L/d*) as seen in **figure 2** of the main paper.

In common with the majority of hydrodynamic studies, the methods in Higdon [3] include three additional simplifying assumptions. First, that sperm have a spherical head, second that the beat of the sperm flagellum is planar and takes a form similar to a sinewave, and finally, that all sperm considered are geometrically similar. However, none of these assumptions is overly restrictive. We take no account of variation in head shape as drag in the low Reynolds number regime at which sperm operate is essentially independent of shape: drag of a prolate spheroid with the length of its major axis equal to the diameter of a spherical head will differ by less than 5% from that of the sphere [5]. If all sperm considered are geometrically similar (especially likely for intraspecific studies) then the effect on velocity will likely be on the slope of the relationship between flagellum and head length, and will produce conclusions that do not differ qualitatively from those given here. Higdon's [3, 4] work applies equally to helical flagellar waves, and similar patterns have been found for other beat patterns [6, 7].

[illegible]

Case (i): Solutions for b = 0, and b = ∞

In both these cases it is helpful to assume that one of the length measures is constant. This is justifiable if the variance of one greatly exceeds that of the other.

If we take L as constant (i.e. when b = 0), then

$$Lt \propto d$$

(A10)

$$U \propto \frac{1}{d}$$

(A11)

and so

$$U \propto \frac{1}{L_t}$$

(A12)

This negative relationship is characterised by increasing steepness as d and L_t diverge (i.e. with increasing difference between d and L_t).

Next, taking d as constant (i.e. when b = ∞) and representing L by the series $[l_1, l_2, \ldots l_n]$, a similar argument to that for constant L gives

$$L_t \propto [l_1, l_2, \ldots l_n]$$

(A13)

$$U \propto \left[l_1/d, l_2/d, \ldots l_n/d \right].$$

(A14)

However, in this case equation (A14) is not reducible, resulting in a positive relationship between U and L_t, the slope of which is < 1, and tends to 0 as d tends to L_t.

Case (ii): Solutions for a range of values of c

Excluding complex-valued solutions the general form in eqns (A4) and (A6) leads to:

$$d = \begin{cases} \frac{1}{2}L_t - \frac{1}{2}a \pm \frac{1}{2}\sqrt{Lt^2 - 2L_t a + a^2 - 4b} & \text{where } c = -1 \\ \frac{\left(-\alpha^{\frac{2}{3}} - 12L_t + 12a\right)^2}{36\alpha^{\frac{2}{3}}} & \text{where } c = -\frac{1}{2} \\ L_t - a - b & \text{where } c = 0 \\ L_t - a + \frac{2b^2}{\beta^{\frac{1}{3}}} - \frac{b\beta^{\frac{1}{3}}}{6} & \text{where } c = \frac{1}{3} \\ L_t - a + \frac{1}{2}b^2 \pm \frac{1}{2}\mathrm{b}\sqrt{b^2 + 4L_t - 4a} & \text{where } c = \frac{1}{2} \\ L_t - a - b\,\frac{\chi^{\frac{2}{3}}}{36} + \frac{4b^4}{9\chi^{\frac{2}{3}}} - \frac{b^2}{9} & \text{where } c = \frac{2}{3} \\ \frac{L_t - a}{b + 1} & \text{where } c = 1 \\ \frac{\delta^{\frac{2}{3}}}{36b^2} + \frac{4}{9b^2\delta^{\frac{2}{3}}} - \frac{1}{9b^2} & \text{where } c = \frac{3}{2} \\ \frac{-\frac{1}{2} \pm \sqrt{1 + 4bL_t - 4ba}}{b} & \text{where } c = 2 \end{cases}$$

where

$$\alpha = -108b + 12\sqrt{-12L_t^{\;3} + 36L_t^{\;2}a - 36L_ta^2 + 12a^3 + 81b^2},$$

(A16)

$$\beta = 108L_t - 108a + 12\sqrt{12b^3 + 81L_t^{\;2} - 162L_ta + 81a^2},$$

(A17)

$$\chi = 108L_t - 108a - 8b^3 + 12\sqrt{81L_t^{\;2} - 162L_ta - 12L_tb^3 + 81a^2 + 12ab^3},$$

(A18)

and

$$\delta = 108b^2L_t - 108b^2a - 8 + 12\sqrt{3}\sqrt{27b^2L_t^{\;2} - 54b^2L_ta - 4L_t + 27b^2a^2 + 4ab}$$

(A19)

The solutions in eqn (A15) can then be rearranged to give U as a function of L_t as in eqn (A9). These are plotted in **figure S2**. Note that If head length increases relative to tail length (scaling factor, c <1) then we expect a negative total length-velocity slope, while if head length decreases relative to tail length (c >1), we expect a positive slope for total length-velocity. When head size decreases relative to tail length, velocity appears to be an asymptotic function of total length, suggesting that increasing sperm length (past a critical point for negative slopes, or for all cases for positive slopes, **figure S2**) will not be detrimental to the sperm's swimming speed.

Table S1. Summary of relationships used in figures.

Taxa	Relationship	Adj. R^2	*P*	Slope	Intercept	Mean head length (μm)	Mean Tail length (μm)	Study
Boar (intraspecific)	n/a*	0.003				8.39		[8]
Atlantic salmon (intraspecific)	Power	0.192	< 0.001	-0.29	3.88			[9]
Mammals	n/a†	-0.008					56.48	[10]
Shorebirds	Linear	0.736	0.0002	2.21	26.92			[11]
Mammals	Power	0.154	< 0.001	0.8	2.62			[12]
Frogs	Linear	0.740	< 0.001	1.51	-4.87			[13]
Frogs	Power	0.301	0.0468	0.46	2.33			[14]
Featherwing beetles	n/a*	-0.235				225.17		[15]

* Variance associated with tail greater than that associated with head.

† Variance associated with head greater than that associated with tail.

Figure S1. Non-dimensional swimming velocity as a function of head length for four values of the non-dimensional amplitude of the tail beat with constant tail length. As the size of the head increases, the influence of wave amplitude decreases. Solid line $\alpha k = 0.5$, dashed line $\alpha k = 1$, dotted line $\alpha k = 2$, dash-dot line $\alpha k = 3$. *Inset*: Original predictions replotted from [3] as non-dimensional amplitude against non-dimensional velocity, illustrating that wave amplitude strongly determines swimming velocity. The four lines represent four values of head/flagellum ratio (from top to bottom: 0, 0.1, 0.2, 0.4). Both figures represent constant length flagellum with a single wave on it [3].

Figure S2. Relationships between total length and speed (normalised by maximum values) given by the solutions set out in the Appendix. Red – c = -1; Black – c = 0; Blue – c = 1/3; Green – c = 1/2; Orange – c = 2/3; Cyan – c = 1; Yellow – c = 1 1/2; Pink – c = 2. Solid and broken lines represent multiple solutions. Colours do not correspond to those in figures 1 and 2.

Figure S1.

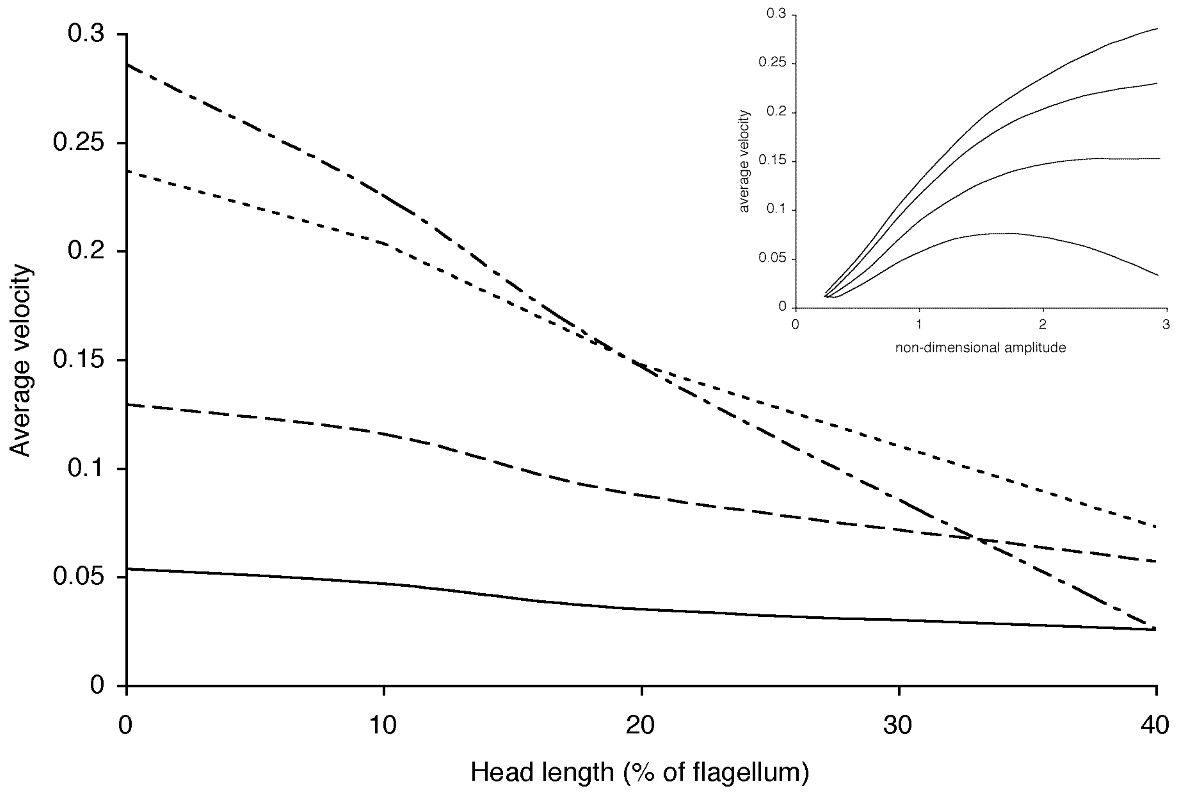

0.3
0.25
0.2
0.15
0.1
0.05
0
Average velocity
0
10
20
30
40
Head length (% of flagellum)
average velocity
non-dimensional amplitude
0
1
2
3

Figure S2.

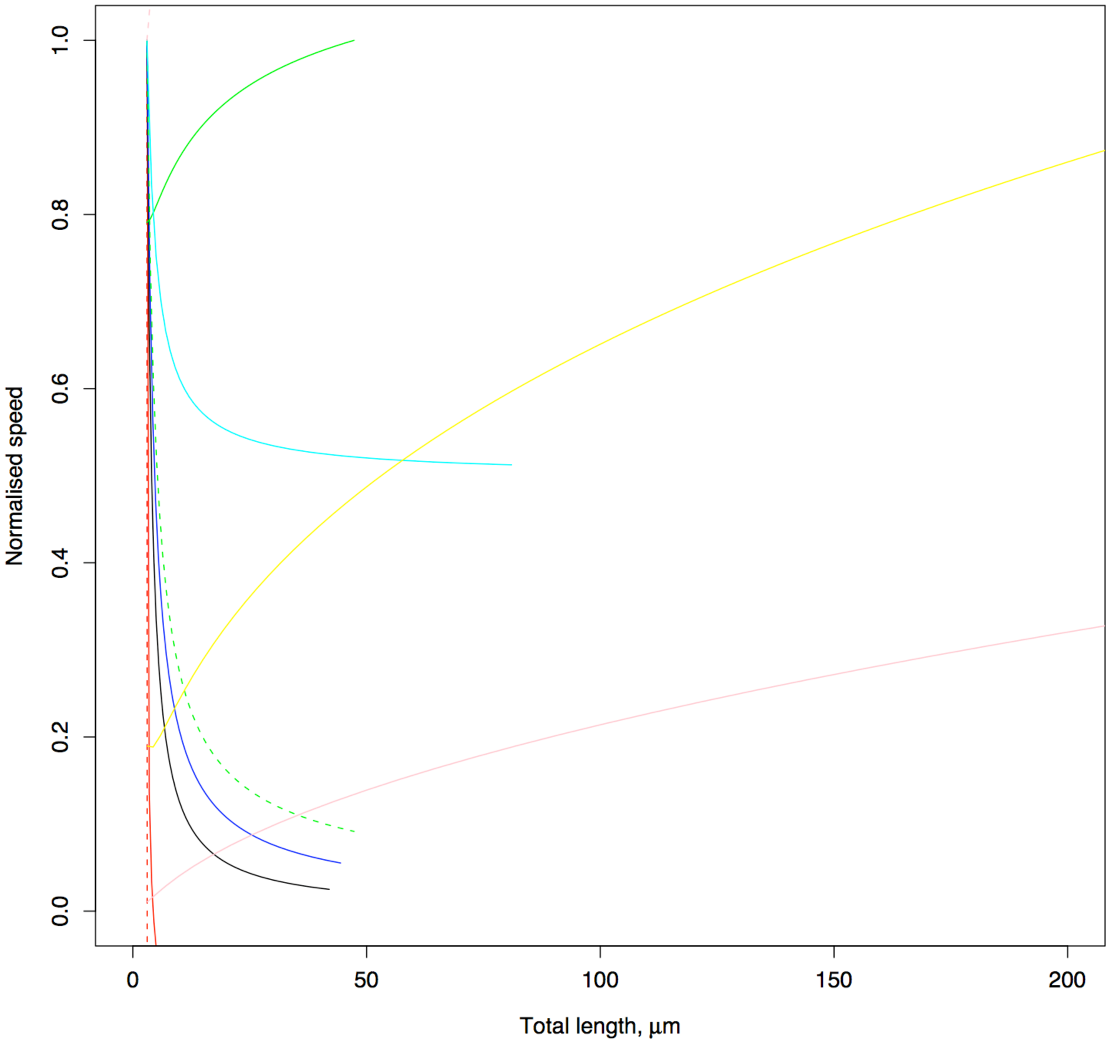

Normalised speed
0.0
0.2
0.4
0.6
0.8
1.0
0
50
100
150
200
Total length, µm

References

1. Brennen C, Winet H: **Fluid mechanics of propulsion by cilia and flagella**. *Ann Rev Fluid Mech* 1977, **9**:339-398.
2. Denny MW: **Air and water: the biology and physics of life's media**. Princeton: Princeton University Press; 1993.
3. Higdon JJL: **A hydrodynamic analysis of flagellar propulsion**. *J Fluid Mech* 1979, **90**(4):685-711.
4. Higdon JJL: **The hydrodynamics of flagellar propulsion: helical waves**. *J Fluid Mech* 1979, **94**(2):331-351.
5. Happel J, Brenner H: **Low Reynolds number hydrodynamics**. Englewood Cliffs, NJ: Prentice-Hall; 1965.
6. Dresdner RD, Katz DF, Berger SA: **The propulsion by large amplitude waves of uniflagellar micro-organisms of finite length**. *J Fluid Mech* 1980, **97**(3):591-621.
7. Johnson RE, Brokaw CJ: **Flagellar hydrodynamics - a comparison between resistive-force theory and slender-body theory**. *Biophys J* 1979, **25**:113-127.
8. Thurston LM, Watson PF, Holt WV: **Sources of variation in the morphological characteristics of sperm subpopulations assessed objectively by a novel automated sperm morphology analysis system**. *J Reprod Fertil* 1999, **117**:271-280.
9. Gage MJG, Macfarlane CP, Yeates S, Shackleton R, Parker GA: **Relationships between sperm morphometry and sperm motility in the Atlantic salmon**. *J Fish Biol* 2002, **61**:1528-1539.

10. Anderson MJ, Nyholt J, Dixson AF: **Sperm competition and the evolution of sperm midpiece volume in mammals**. *J Zool* 2005, **267**:135-142.

11. Johnson DDP, Briskie JV: **Sperm competition and sperm length in shorebirds**. *Condor* 1999, **101**(4):848-854.

12. Gage MJG: **Mammalian sperm morphometry**. *Proc Roy Soc Lond B* 1998, **265**:97-103.

13. Byrne PG, Simmons LW, Roberts JD: **Sperm competition and the evolution of gamete morphology in frogs**. *Proc Roy Soc Lond B* 2003, **270**:2079-2086.

14. Kuramoto M, Joshy HS: **Scanning electron microscopic studies on spermatozoa of anurans from India and Sri Lanka**. *Amphib Reptil* 2004, **22**:303-308.

15. Dybas LK, Dybas HS: **Coadaptation and taxonomic differentiation of sperm and sperathecae in featherwing beetles**. *Evolution* 1981, **35**(1):168-174.
